# Developing a digital health competency assessment framework for public health services: a Delphi-AHP approach

**DOI:** 10.3389/fpubh.2025.1634261

**Published:** 2025-07-21

**Authors:** Yibei Yao, Jindan Cao, Zheng Tan, Keli Chen

**Affiliations:** ^1^School of Public Health, Jilin University, Changchun, China; ^2^Shenzhen Health Development Research and Data Management Centre, Shenzhen, China

**Keywords:** digital health competency, public health, indicator system, Delphi method, analytic hierarchy process

## Abstract

**Objective:**

To construct a scientifically validated and practical digital health competency assessment framework for public health services, addressing gaps in current evaluation paradigms that fail to comprehensively assess individuals’ proactive engagement with digital health tools.

**Methods:**

The study employed a mixed-methods approach, beginning with a systematic literature review and structured interviews to draft an initial indicator system. Two rounds of Delphi consultations with 15 experts were conducted to refine the framework, followed by quantification of indicators using the Analytic Hierarchy Process (AHP).

**Results:**

The final framework comprises 3 primary indicators (Health Literacy, Information Literacy, Digital Competency), 9 secondary indicators, and 19 tertiary indicators. Health Literacy and Information Literacy were weighted highest (0.4545 each), reflecting their foundational roles, while Digital Competency (0.0909) served as a complementary enabler.

**Conclusion:**

This study addresses a critical gap in digital health research by providing a multidimensional assessment tool that integrates technological, cognitive, and behavioral dimensions. The framework aligns with global digital health strategies and responds to societal challenges like the “digital divide.” Its practical applications include guiding policy-making, optimizing digital health services, and tailoring health education programs to improve public health outcomes.

## 1 Introduction

Under the continuous advancement of the scientific and technological revolution and industrial change, digital technology is gradually injecting new momentum into the healthcare industry, driving the traditional healthcare service system to digitization and intelligentization (1). Digital health as a specific form of new productivity in the medical field, is continuing to break through the boundaries of traditional medical production possibilities through the spiral of “technological innovation, institutional innovation and business innovation.” The use of digital technology to improve health, innovate service products and models, and promote the sustainable development of digital health has become a key element of healthcare policy. Driven by this trend, the concept of digital health has gradually taken shape and gained widespread attention (2). In “the Global Strategy for Digital Health (2020–2025)” published by the World Health Organization (WHO) states that digital health refers to the theoretical systems and practical approaches that utilize various digital technologies to improve health. This concept not only includes the development and application of intelligent terminal devices, but also involves research on the behavior of consumers of digital healthcare services, as well as the innovative practice of integrating and applying cutting-edge technologies such as the Internet of Things (IoT), artificial intelligence (AI), cloud computing, big data analytics, and intelligent robots to healthcare service systems (3).

Effective public health management and health service system development in the digital health landscape must be grounded in citizens’ health literacy and information literacy (4), supported by digital technologies to guide the citizens in reshaping health perceptions and enhancing health management and decision-making capabilities (5). Health literacy is the ability of an individual to access, understand, evaluate, and apply health information to make appropriate health decisions (6). Studies have shown that higher levels of health literacy are associated with better disease prognosis (7). Information literacy, on the other hand, is characterized by an individual’s ability to use information, including the ability to perceive, comprehend, identify, and apply it (8). In public health events, information literacy directly affects the people’s understanding of and cooperation with professional guidance (9). However, the persistence of the “digital divide”--the differences in access to, ability to use, and effectiveness of digital technologies among different groups (10), has led to significant inequalities in the use of digital tools by individuals to access health information and process health content (11). This divide is particularly pronounced in groups such as older adults and those living in rural areas (12).

Current assessment paradigms relying solely on digital competency, health literacy, or health information literacy have proven inadequate for comprehensively evaluating individuals’ proactive engagement with health-related media (13). This underscores the urgent need to develop a multidimensional assessment framework that captures the full spectrum of public competencies in digital health contexts (14).

Scholarly research on public digital health competency remains nascent, particularly regarding the establishment of systematic assessment frameworks with standardized, scientifically validated indicators (15). This study aims to construct a comprehensive, scientifically robust, and operationally feasible assessment indicator system for public digital health competency. Through systematic review of domestic and international literature coupled with analysis of public health needs and digital technology adoption patterns, we develop a multidimensional indicator system. The framework’s scientific validity and practical utility are ensured through Delphi expert consultation and Analytic Hierarchy Process (AHP) methodology (16).

The resulting assessment system provides critical support for:

1. Improving the digital health competency of the public health users;
2. Promoting the widespread adoption and development of digital health services;
3. Informing policy-making for equitable digital health integration.

## 2 Methods

### 2.1 Indicator selection

#### 2.1.1 Literature review

A systematic literature search was conducted using key terms including “health literacy, digital literacy, information literacy, e-health literacy, health information literacy, digital competency, media literacy” in the Web of Science Core Collection and PubMed databases. Sixty-nine documents closely related to this study were finally included through manual screening. Policy documents closely related to digital health capacity included in this study after intra-group discussion and screening, including the UNESCO’s “Global Digital Literacy Skills Reference Framework for Indicator 4.4.2” (17), the World Health Organization’s “Global Digital Health Strategy 2020–2025” (18), the European Union’s “Digital Competence Framework (DigComp 2.2)” (19), the DQ Institute’ s Digital Intelligence (DQ) Framework (20). Comprehensively analyze the literature and policy documents related to digital health capacity, and form an initial indicator system of digital health capacity after discussion, reform, and screening according to the principle of building an indicator system.

#### 2.1.2 Qualitative research

Semi-structured interviews were conducted via offline face-to-face and online meeting formats with 10 frontline healthcare professionals and 5 researchers specializing in digital healthcare with more than 5 years of experience. The results of the interviews were used to validate and refine the indicators initially screened from the literature review. The interview questions included:

1. How do you perceive the distinctions and connections between digital health competency and traditional health literacy?
2. What do you consider the core components of digital health competency?
3. Which dimensions should be prioritized when assessing digital health competency?
4. Do you have any recommendations or insights regarding the construction of an assessment indicator system for digital health competency?

### 2.2 Expert selection

The selection of Delphi experts should be based on the knowledge domain requirements of the research topic. The panel size should be determined by considering both the project scale and disciplinary coverage, research suggests that a minimum of 12 panellists are required to achieve a consensus (21). The inclusion criteria for experts in this study were as follows:

1. Possession of at least a bachelor’s degree and intermediate-level professional technical qualifications;
2. Minimum 5 years of practical experience in relevant specialized fields;
3. Demonstrated domain-specific expertise capable of providing valuable recommendations;
4. Voluntary participation in the study;
5. commitment to complete all consultation rounds.

Accordingly, this study engaged 15 authoritative experts from relevant fields to participate in the consultation process.

### 2.3 Questionnaire design

The questionnaire comprised four sections:

1. Introduction: Background and objectives of the consultation, instructions for questionnaire return, contact information of experts, and acknowledgment.
2. Digital Health Competency Assessment Indicator Evaluation Form: Included conceptual definitions of digital health Competency, detailed instructions, and a consultation table for tertiary indicators.
3. Expert Information Survey: Collected demographic data (e.g., professional field, technical title, contact details).
4. Expert Familiarity and Judgment Basis Survey: Assessed experts’ cognitive level of the consultation content, including theoretical foundations and practical experience applied during evaluation.

### 2.4 Delphi method

The importance of each content was classified into five levels of very unimportant-very important according to the Likert5 classification, assigned a score of 1–5 respectively, and the experts scored the importance of each content according to their own judgement and made suggestions for modification, deletion or addition. Expert motivation was quantitatively assessed by the effective recovery rate of the questionnaire. Expert authority coefficient (*Cr*) indicates the average of the judgment criteria of expert judgment basis (*Ca*) and familiarity with the content of the indicator (*Cs*). The basis of expert judgment (*Ca*) is calculated through four dimensions: theoretical analysis, practical foundation, reference materials and intuitive judgment. The score of theoretical analysis is 0.1–0.3, the score of practical foundation is 0.3–0.5, the score of reference materials and intuitive judgment is 0.1, and the influence of each dimension on the basis of expert judgment is divided into three grades: large, medium and small. Expert familiarity (*Cs*) was assessed using a 5-point Likert scale, with assigned values of 0.2, 0.4, 0.6, 0.8, and 1.0 from “very unfamiliar” to “very familiar,” respectively. When *Cr* ≥0.7, the expert’s consulting results are considered to have a high degree of credibility (22). The consistency of expert opinions can be evaluated by the coefficient of variation (*Cv*) and *Kendall’s W*, in which *Cv* < 0.25 indicates that the degree of dispersion of expert ratings is low and the results are relatively stable; the value of *W* ranges from 0 to 1, and the larger the value, the better the concordance of the expert’s opinions is reflected, and *W* ≥ 0.5 is regarded as high correlation. If the *p* value of *Kendall’s W* test is less than 0.05, it indicates that the expert opinions are statistically consistent (23).

The screening conditions for the indicators in this study were as follows: (1) Items that met the criteria of mean score ≥4 and coefficient of variation <0.25 were retained (24). (2) If more than 30% of the experts rated an indicator as ‘not necessary’ the indicator was censored (25). (3) If the dissenting opinion was less than 30% but the arithmetic mean of the experts’ ratings for the indicator was less than 3.5 or the coefficient of variation was less than 0.5, the indicator was censored. 3.5 or the coefficient of variation (*CV*) is greater than 0.3, the reasonableness of the indicator will be further argued and the redundant or non-critical indicators will be deleted according to the results of the argumentation. (4) If the experts suggest new indicators, the necessity should be assessed in the light of the literature, and the indicators that are really necessary should be included in the next round of expert consultation. (5) Expert consultation on the revised indicators will be conducted again until the experts’ opinions converge, that is, to stop the correspondence.

### 2.5 AHP method

The Analytic Hierarchy Process (AHP) is a multi-criteria decision-making method that quantifies qualitative problems (26). This method calculates hierarchical weights and rankings by decomposing elements related to decision-making into a number of objectives and levels, and calculating hierarchical weights and rankings by fuzzy quantification of qualitative indicators, in order to realize a quantitative decision-making method. In this study, the Delphi method was combined with the hierarchical analysis method (27). A hierarchical model was established based on the digital health competency indicators determined from the results of the 2 rounds of Delphi expert correspondence, and then a judgement matrix was constructed from the mean difference of the digital health competency indicators in the expert consultation, and the consistency test of the matrix was performed using the Yaahp12.9 software, with a ratio of less than 0.1 indicating that a consistency test had been passed (28), the weights of indicators as well as the combination weights are calculated and the combination weights are ranked.

### 2.6 Statistical methods

This study mainly used the application of Excel to create a database, and SPSS 26.0 software was used to analyze and calculate the data for the mean, coefficient of variation, coefficient of expert positivity, coefficient of expert authority, and degree of expert coordination.

## 3 Results

### 3.1 Basic information on experts

In this study, the Delphi method was used to carry out two rounds of expert consultation, and the questionnaire recovery rate of both rounds of survey reached 100%, indicating that the experts’ participation was good and the research data had a high degree of reliability. The experts’ academic qualifications were all bachelor’s degree or above, of which 86.7% were postgraduate degrees, and 86.7% were experts with deputy senior and above titles. The authority coefficient (Cr) of the experts is 0.739, which is more than 0.70, proving their high authority and prediction accuracy. As the indicator shows a CV of less than 25.0%, the experts’ opinions indicate little fluctuation in the content of the study. Kendall’s consistency coefficient is statistically significant in both rounds of consultation, indicating a high degree of consensus among the experts on the content of the consultation.

### 3.2 Degree of coordination of expert advice

The coefficients of variation for the first round of expert correspondence were 0.00 to 0.43, and the Kendall’s coefficient of concordance for all entries in the first round *W* = 0.292 (*x^2^* = 196.204, *p* < 0.05), which indicated a considerable degree of consistency. The coefficient of variation of the second round of expert correspondence was 0.00 ~ 0.19, and the Kendall coordination coefficient of all the entries in the second round *W* = 0.514 (*x^2^* = 254.174, *P*<0.05), with a *W* value of more than 0.5, indicating a higher degree of consistency and a better control of errors. The results show that after two rounds of Delphi expert consultation, the degree of coordination of the expert group’s opinions is significantly higher (the coefficient of variation is lower), and the level of consistency of the evaluation indicators is higher, which indicates that the experts have a good degree of acceptance of the indicator system, and the results of the study have a good degree of reliability.

### 3.3 Digital health competency indicator framework

Indicator elements were screened, and relevant indicators were revised in conjunction with expert feedback. In the end, a total of three indicators (digital identity management, digital protection and digital participation) were excluded from the first round of the survey, and two were revised (combining “emotion and communication” and “digital protection” into “digital competency”; and revising “health problem solving” into “health literacy”). After the second round of survey, one indicator (digital security) was deleted and one was revised (from “internet etiquette” to “digital ethics and identity”), finally forming the public digital health competency indicator framework. The indicator system covers 3 primary indicators, 9 secondary indicators and 19 tertiary indicators. Primary indicators represent the broad dimensions of digital health competency. Secondary indicators break down each primary dimension into specific competency areas. Tertiary indicators further detail the specific skills or behaviors within each secondary competency. The specific evaluation indicators and their explanations are shown in Table 1.

**Table 1:** Indicators for the evaluation of digital health competency and their interpretation.

	Indicator name	Interpretation of indicators
Primary indicators	1. Health Literacy	Health literacy is needed by the public when seeking help with health in digital environments, including identifying health needs, making health decisions, and using decisions to promote health behavior change.
	2. Information literacy	Information literacy for the public seeking help with health in the digital environment, covering browsing, searching, evaluating and managing health information.
	3. Digital competency	Digital competency for the public seeking help with health in the digital environment, including digital interaction, digital emotions, digital safety and digital health protection.
Secondary indicators	1.1 Health needs expression competency	The competency of the public to be aware of their health needs and to accurately assess and express them in a data environment.
	1.2 Health decision making competency	The competency to use digital tools and technologies in a data environment to make sound health decisions based on acquired health data, information and knowledge.
	1.3 Health behavior change competency	Competency of the public to use health decision-making to promote health behavior change.
	2.1 Health information acquisition competency	The competency of the public to search for health data, information, and content in the digital environment, access them, and be able to create and update personal search strategies.
	2.2 Health information screening competency	The competency of the public to analyze, compare and critically assess the credibility and reliability of sources of health data, information and digital content in the digital environment.
	2.3 Health information management competency	The competency to store and organize health data, information and content in a digital environment.
	3.1 Digital interaction competency	The competency to use digital tools and technologies in the digital environment for communication and interaction on health-related issues, as well as for the co-construction and co-creation of health resources and knowledge.
	3.2 Digital emotional competency	The competency to recognize, understand and manage their own and others’ emotions in a digital environment. This includes demonstrating empathy and inclusiveness in online interactions related to health issues.
	3.3 Digital protection competency	The competency to protect their own and others’ personal information, digital media, and physical and mental health when seeking help for health in a digital environment.
Tertiary indicators	1.1.1 Health problem assessment	The public can accurately assess health problems when seeking health help in the digital environment.
	1.1.2 Health needs expression	The public can accurately explain and express health information needs when seeking help with health in a digital environment.
	1.2.1 Personalized health decision making	The public can use health information to make personalized health decisions when seeking health help in a digital environment.
	1.3.1 Adverse health behavior change	The public can use personalized health decisions to change poor health behaviors.
	2.1.1 Search tool selection	The public should be able to choose appropriate search tools when seeking health help in digital environments.
	2.1.2 Search strategy construction	The public can use appropriate search language and search form when seeking help for health in the digital environment.
	2.2.1 Health information screening	The public can reason critically and evaluate information and data in the digital environment.
	2.2.2 Health information reasoning evaluation	The public can assess the reliability and authority of health information content through information sources in the digital environment.
	2.2.3 Health information comparative evaluation	The public can judge the correctness of information by comparing different information when seeking health help in the digital environment.
	2.3.1 Health information preservation/collection	The public can save/collect health information in the digital environment.
	2.3.2 Health information organization	The public can classify and organize health information in the digital environment.
	3.1.1 Health activity participation	The public can use public and private digital services to participate in community health activities online in the digital environment.
	3.1.2 Health data sharing	The public can share health data, information and digital content with others using appropriate digital technologies in the digital environment.
	3.1.3 Health problem discussion	The public can participate in discussions on health-related issues through digital and online platforms in the digital environment to support the formation of sound health decisions
	3.2.1 Emotion recognition and expression	The public can understand and recognize their own and others’ health needs, feelings and concerns online in the digital environment.
	3.2.2 Digital ethics and identity	The public is able to adopt proper online etiquette and have a sense of morality and acceptance of digital ethics when seeking help for their health in the digital environment.
	3.3.1 Information protection	The public is able to avoid intentional or unintentional theft of important personal information or data in the process of transmission, storage and use in the digital environment.
	3.3.2 Digital media protection	When seeking health assistance in the digital environment, the public can recognize whether digital media are exposed to cyber threats (viruses, malware) and can take proper security precautions.
	3.3.3 Physical and mental health protection	When seeking health help in the digital environment, the public can avoid possible risks in the digital environment (e.g., cyberbullying) and protect their own mental health and that of others.

### 3.4 Importance weighting of digital health competency indicators

The consistency ratios of the judgement matrices of the first level indicators and the three second level indicator judgement matrices and the nine third level indicator judgement matrices in this study were 0.0000, 0.0000, 0.0633, 0.0089, 0.0000, 0.0000, 0.0000, 0.0000, 0.0517, 0.0000, 0.0176, 0.0000, 0.0000, 0.0000, respectively, 0.0000 whose results are all less than 0.1, passing the consistency test. Table 2 shows the importance assignment and weights of public digital health competency assessment indicators.

1 Primary indicators weights

**Table 2:** Importance assignment and weighting of digital health competency assessment indicators.

Indicators	Importance assignment		Weights
	score ( $\bar{x}\pm s$ )	*Cv*	
1 Health literacy	5.00 ± 0.00	0.00	0.4545
1.1 Health needs expression competency	5.00 ± 0.00	0.00	0.2453
1.1.1 Health problem assessment	5.00 ± 0.00	0.00	0.1635
1.1.2 Health needs expression	4.87 ± 0.34	0.07	0.0818
1.2 Health decision making competency	4.87 ± 0.34	0.07	0.1350
1.2.1Personalised health decision making	4.80 ± 0.40	0.08	0.1350
1.3 Health behavior change competency	4.80 ± 0.40	0.08	0.0743
1.3.1 Adverse health behavior change	4.67 ± 0.47	0.10	0.0743
2 Information literacy	5.00 ± 0.00	0.00	0.4545
2.1 Health information acquisition competency	4.93 ± 0.25	0.05	0.2121
2.1.1 Search tool selection	4.40 ± 0.61	0.14	0.1591
2.1.2 Search strategy construction	4.20 ± 0.65	0.16	0.0530
2.2 Health information screening competency	4.93 ± 0.25	0.05	0.2121
2.2.1 Health information screening	4.87 ± 0.34	0.07	0.0530
2.2.2 Health information reasoning evaluation	4.93 ± 0.25	0.05	0.1061
2.2.3 Health information comparative evaluation	4.87 ± 0.34	0.07	0.0530
2.3 Health information management competency	4.00 ± 0.52	0.11	0.0303
2.3.1 Health information preservation/collection	4.07 ± 0.57	0.14	0.0202
2.3.2 Health information organization	4.00 ± 0.52	0.13	0.0101
3 Digital competency	4.33 ± 0.47	0.11	0.0909
3.1 Digital interaction competency	4.13 ± 0.81	0.19	0.0171
3.1.1 Health activity participation	4.27 ± 0.68	0.16	0.0085
3.1.2 Health data sharing	4.20 ± 0.40	0.10	0.0053
3.1.3 Health problem discussion	4.13 ± 0.62	0.15	0.0034
3.2 Digital emotional competency	3.80 ± 0.65	0.17	0.0074
3.2.1 Health problem discussion	4.00 ± 0.63	0.16	0.0055
3.2.2 Digital ethics and identity	3.80 ± 0.54	0.14	0.0018
3.3 Digital protection competency	4.73 ± 0.57	0.12	0.0664
3.3.1 Personal information protection	4.60 ± 0.49	0.11	0.0371
3.3.2 Digital media protection	4.47 ± 0.50	0.11	0.0212
3.3.3 Physical and mental health protection	4.20 ± 0.40	0.10	0.0081

The primary indicators of the public digital health competency indicator system constructed in this study include three items, the weights of the three dimensions are: health literacy (0.4545), information literacy (0.4545), and digital competency (0.0909).

2 Secondary indicator weights

The digital health competency indicator system constructed in this study includes a total of 9 secondary indicators. Among them, there are 3 s-level indicators under the first-level indicator Health Literacy dimension, namely, the ability to express health needs (0.2450), the ability to make health decisions (0.1351), and the ability to change health behaviors (0.0744); 3 secondary indicators under the information literacy dimension of the first-level indicator, health information acquisition ability (0.2121), health information screening ability (0.2121), and health information management ability (0.0303); the digital competency dimension of the first-level indicator has 3 items of digital interaction ability (0.0176), digital emotional ability (0.0076), and digital protection ability (0.0658) Second-level indicators.

3 Tertiary indicator weights

The digital health competency indicator system constructed in this study includes a total of 19 tertiary indicators. In the dimension of health literacy, the ability to express health needs includes 2 indicators under the secondary indicator of the ability to assess health problems (0.1633) and express health needs (0.0817). The second-level indicator of health decision-making ability includes 1 indicator of personalized health decision-making (0.1351). Under the secondary indicator of health behavior change ability, there is 1 indicator of adverse health behavior change (0.0744). In the digital literacy dimension, 3 indicators were included under the secondary indicator of digital interaction ability, including health activity participation (0.0086), health data sharing (0.0055), and health problem discussion (0.0035). In the information literacy dimension, 2 indicators were included under the second-level indicator of health information acquisition ability, including the selection of retrieval tools (0.1591) and the construction of retrieval strategies (0.0530). Under the second-level indicator of health information screening ability, there are 3 indicators including health information reasoning evaluation (0.1061), information source screening (0.0530), and health information comparison (0.0530). Under the secondary indicator of health information management ability, there are 2 indicators including health information preservation and collection (0.0202), and health information organization (0.0101). Under the secondary indicator of digital emotional competency, there are 2 indicators including emotion recognition and expression (0.0057), and digital ethical and moral identity (0.0019). Under the secondary indicator of digital protection ability, there are 3 indicators including personal information protection (0.0366), digital media protection (0.0211), and physical and mental health protection (0.0081).

## 4 Discussion

### 4.1 Analysis of the scientific validity and practical value of the indicator system

The digital health competency assessment indicator system of this study has high scientific validity and practicality. In terms of scientific validity, the positive coefficient of experts in this study is high, and the recovery rate of questionnaires in both rounds is 100%, indicating that it reflects the great importance and continuous attention of the expert group members to the topic of this study. The coefficient of expert authority (*Cr*) was 0.739, indicating that the experts’ academic level was high and their judgement was well grounded, and they were more familiar with areas related to public digital health competency. In addition, this study assigns weights to the indicators based on the hierarchical analysis method, which further improves the scientificity and rationality of the indicator system construction.

In terms of practicality, the indicator system constructed in this study has certain practical value. The indicator system consists of three primary indicators, nine secondary indicators and 19 tertiary indicators. The relationship between health literacy, information literacy, digital competency and digital health competency is multidimensional, in which each competency domain of health literacy, information literacy, digital competency may affect one or more competency domains of digital health competency (29) but may all fail to cover certain competencies of digital health competency (30). The health literacy dimension consists of three secondary indicators, namely, the ability to express health needs, the ability to make health decisions, and the ability to change health behaviors. By evaluating these abilities, we can systematically measure an individual’s comprehensive literacy level in the acquisition of health information, optimization of health behaviors, and coping with health risks, which will provide a scientific basis for the relevant authorities to formulate precise health intervention strategies (31). In the information literacy dimension, health information acquisition competency (32), health information screening competency (33), and health information management competency (34) are key competencies for the public to acquire and process health information, and the assessment of these competencies can help to systematically examine the public’s comprehensive competencies in health information resource retrieval, information authenticity recognition, and information integration and application. Among the digital competency dimensions, digital interaction competency, digital emotional competency, and digital protection competency are important supports for the public to use health information in the digital environment (35, 36). The assessment of these abilities can objectively measure an individual’s digital competency in the process of health information processing, and provide a key basis for optimizing the effectiveness of digital health services.

### 4.2 Analysis of the rationality of the allocation of weights to different dimensions in the indicator system

The study assigns weights to the indicators in the digital health competency assessment system based on the hierarchical analysis method, in which the weights of the first-level indicators of health literacy and information literacy are both 0.4545, and the weight of digital competency is 0.0909. This weighting indicates that in the current digital health competency assessment framework, public health cognitive ability and information processing ability dominate the digital health competency assessment framework, while the digital competency plays an indispensable auxiliary role, digital competency plays an indispensable auxiliary role. The result reflects that the development of digital health competency at this stage is still in a transitional stage centred on traditional health literacy and gradually transforming to digital. The reasonableness of the result is reflected in the following aspects:

The core status of Health Literacy. Health literacy can be defined as “the extent to which an individual is able to obtain, process, understand, and communicate health-related information needed to make informed health decisions” (37). On 30 December 2015, the Basic Knowledge and Skills for Chinese Citizens” Health Literacy (2015 Edition) issued by the former Health Planning Commission referred to health literacy and defined it as the ability of an individual to obtain, understand, and communicate health-related information. as an individual’s ability to acquire, understand, screen, and apply health information. Public health literacy is closely related to health-related quality of life (HRQoL). Patients with higher levels of health literacy tend to have a more favorable disease prognosis, and this group is able to improve disease progression through improved self-management (38). Low health literacy is associated with a higher prevalence of chronic disease, with individuals in the low health literacy group being 1.33 times more likely to develop chronic disease compared to those with high health literacy (39). Individuals with multiple chronic diseases tend to have lower health literacy scores, suggesting a relationship between the number of chronic diseases and the level of health literacy. Number of chronic diseases and the level of health literacy (40). Health literacy directly and positively predicts the health-related quality of life of the population (41). Furthermore, improving health literacy among individuals with chronic diseases is an important pathway towards active health. These findings emphasize the critical role of health literacy in improving health management and quality of life for people with chronic diseases.

The importance of information literacy is growing. Information literacy is the ability to reflect on and discover information, understand the generation and evaluation of information, use information to create new knowledge, and participate in learning communities. High information literacy can help the public to effectively identify the authenticity of health information and avoid being misled by false health information (42). Studies have shown that individuals with higher levels of information literacy perform better in terms of the quality of health decision-making, awareness of disease prevention, and efficiency in the use of healthcare resources (43). Particularly in the case of public health emergencies, the level of information literacy directly determines the extent to which the public understands professional guidance and cooperates with responses. Information literacy is a key element in the quality of life. Measures (44).

The complementary role of digital competency. Digital competency plays a major role in the field of health promotion as a technological enabler. Digital competency can be thought of as an umbrella term for many different technologies and affects various areas of human life, such as education, business, health, and governance. It has been included in the recommendations on key competences for lifelong learning proposed by the European Commission as one of the eight key life skills, and it has been defined as (45) “the confident, critical and responsible use of, and engagement with, digital technologies for learning, at work, and for participation in society.” Digital competency is the public’s overall ability to function in a digital environment (46). It encompasses a wide range of aspects such as access to digital information, evaluation, management, and security awareness, and it is a way to safeguard the needs of a digital life, enjoy the convenience of digital society, and the basic ability to integrate into digital society (47).

In summary, the public digital health competency assessment indicator system constructed in this study is reasonable in terms of weight allocation and can accurately reflect the core components of public digital health competency. This weighting not only provides a scientific basis for assessing the public’s digital health competency, but also provides an important reference for formulating targeted health education strategies and optimizing digital health services.

### 4.3 The significance of constructing a public digital health competency assessment indicator system

Against the backdrop of the wave of digitization sweeping the world, digital health services have become an important way to improve the health of the entire population. However, the existence of the digital divide and the explosive growth of health information have resulted in some groups being hindered in accessing and utilizing digital health resources (48). This study constructs a digital health competency assessment indicator system to systematically sort out and integrate the multidimensional connotations of digital health competency, and proposes that in order to achieve substantial improvement in the public’s health decision-making ability, it is necessary to establish a three-dimensional synergistic digital competency with health literacy and information literacy mechanism. Health literacy provides the basis for professional cognition, information literacy ensures the effectiveness of information processing, and digital competency provides the path for technical realization. The three key competencies are interdependent and dynamically synergistic, and together they constitute the “golden triangle” support system for public digital health competency.

Digital health industry is a new engine for the development of the future health industry, and its development cannot be separated from public participation and support. By assessing the digital health competency, health education and digital technology popularization strategies can be formulated in a targeted manner, thereby improving the public’s digital health competency and promoting changes in health behaviors. In addition, the indicator system can also provide scientific reference for the government and relevant organizations to formulate digital health policies and optimize digital health services.

The digital health competency assessment indicator system is an important cornerstone for promoting the development of digital health. By constructing and improving the system, it can effectively enhance public digital health competency, promote the high-quality development of the digital health industry, and ultimately achieve the goal of universal health. In the future, the project team intends to verify the validity of the indicator system through large-sample empirical research, improve the scientificity and practicality of the digital health competency assessment, and provide a stronger guarantee for the implementation of the Global Strategy on Digital Health.

## Data Availability

The raw data supporting the conclusions of this article will be made available by the authors, without undue reservation.

## References

[1] Gopal G Suter-Crazzolara C Toldo L Eberhardt W (2019). Digital transformation in healthcare - architectures of present and future information technologies. Clin Chem Lab Med 57:328–35. doi: 10.1515/cclm-2018-0658, PMID: 30530878

[2] Yeung AWK Torkamani A Butte AJ Glicksberg BS Schuller B Rodriguez B (2023). The promise of digital healthcare technologies. Front Public Health 11:1196596. doi: 10.3389/fpubh.2023.1196596, PMID: 37822534 PMC10562722

[3] Cingolani M Scendoni R Fedeli P Cembrani F (2022). Artificial intelligence and digital medicine for integrated home care services in Italy: opportunities and limits. Front Public Health 10:1095001. doi: 10.3389/fpubh.2022.1095001, PMID: 36684935 PMC9849776

[4] Busse TS Nitsche J Kernebeck S Jux C Weitz J Ehlers JP (2022). Approaches to improvement of digital health literacy (eHL) in the context of person-centered care. Int J Environ Res Public Health 19:8309. doi: 10.3390/ijerph19148309, PMID: 35886158 PMC9316109

[5] Mäder M Schönfelder T Heinrich R Militzer-Horstmann C Timpel P (2025). Effectiveness of digital health applications on the quality of life in patients with overweight or obesity: a systematic review. Arch Public Health 83:3. doi: 10.1186/s13690-024-01474-3 39780228 PMC11715991

[6] Nengomasha CT Abankwah RM Uutoni WE Pazvakawambwa L. Health information literacy of the University of Namibia’s students. J Stud Humanit Soc Sci. (2015) 4:179–92. Availavle at: https://research.ebsco.com/linkprocessor/plink?id=f64a401e-6b09-3b1d-94c8-3403a038ba81

[7] Chen S Bai Q Zhu J Liu G (2025). Impact of functional, communicative, critical and distributed health literacy on self-management behaviors in chronic disease patients across socioeconomic groups. BMC Public Health 25:1776. doi: 10.1186/s12889-025-23003-9, PMID: 40369489 PMC12076823

[8] Sharma SL S I (2013). Information literacy among faculty and students of postgraduate institute of medical education and research, Chandigarh and Pt. B. D. Sharma university of health sciences, Rohtak. Int J Inf Dissem Technol 3:244–8. Available at: https://www.ijidt.com/index.php/ijidt/article/view/3.4.4

[9] Eysenbach G How to fight an infodemic: the four pillars of infodemic management. J Med Internet Res. (2020) 22:e21820. doi: 10.2196/21820 32589589 PMC7332253

[10] Western MJ Smit ES Gültzow T Neter E Sniehotta FF Malkowski OS (2025). Bridging the digital health divide: a narrative review of the causes, implications, and solutions for digital health inequalities. Health Psychol Behav Med 13:2493139. doi: 10.1080/21642850.2025.2493139, PMID: 40276490 PMC12020140

[11] Estacio EV Whittle R Protheroe J (2019). The digital divide: examining socio-demographic factors associated with health literacy, access and use of internet to seek health information. J Health Psychol 24:1668–75. doi: 10.1177/1359105317695429, PMID: 28810415

[12] Diana MG Mascia ML Tomczyk Ł Penna MP (2025). The digital divide and the elderly: how urban and rural realities shape well-being and social inclusion in the Sardinian context. Sustain For 17:1718. doi: 10.3390/su17041718

[13] Kemp E Trigg J Beatty L Christensen C Dhillon HM Maeder A (2021). Health literacy, digital health literacy and the implementation of digital health technologies in cancer care: the need for a strategic approach. Health Promot J Aust 32:104–14. doi: 10.1002/hpja.387, PMID: 32681656

[14] Thorup CB Uitto M Butler-Henderson K Wamala-Andersson S Hoffrén-Mikkola M Schack Thoft D (2025). Choosing the best digital health literacy measure for research: mixed methods study. J Med Internet Res 27:e59807. doi: 10.2196/59807, PMID: 40198098 PMC12015337

[15] Ji H Dong J Pan W Yu Y (2024). Associations between digital literacy, health literacy, and digital health behaviors among rural residents: evidence from Zhejiang, China. Int J Equity Health 23:68. doi: 10.1186/s12939-024-02150-2, PMID: 38594723 PMC11003150

[16] Jiang N Liu WB Zong Y Yu L Cheng S (2024). Construction of an index system of core competence assessment for sleep medicine nurse specialists in China: a delphi study. BMC Nurs 23:689. doi: 10.1186/s12912-024-02349-2, PMID: 39334099 PMC11438144

[17] A global framework of reference on digital literacy skills for indicator 4.4.2 - UNESCO digital library. Available online at: https://unesdoc.unesco.org/ark:/48223/pf0000265403 (Accessed June 24, 2025)

[18] Global strategy on digital health 2020-2025. Available online at: https://www.who.int/publications/i/item/9789240020924 (Accessed June 24, 2025)

[19] Digcomp: the EU digital competence framework | digital skills & jobs platform. Available online at: https://digital-skills-jobs.europa.eu/en/actions/european-initiatives/digital-competence-framework-digcomp (Accessed June 24, 2025)

[20] Digital literacy – DQ institute. Available online at; https://www.dqinstitute.org/global-standards/ (Accessed June 24, 2025)

[21] Vogel C Zwolinsky S Griffiths C Hobbs M Henderson E Wilkins E (2019). A Delphi study to build consensus on the definition and use of big data in obesity research. Int J Obes 43:2573–86. doi: 10.1038/s41366-018-0313-9, PMID: 30655580 PMC6892733

[22] Fu J Jin L Shang X Zhang C Chen L (2025). Construction of a home-based self-management program for patients with indwelling peripherally inserted central catheter based on the IMB model. BMC Nurs 24:433. doi: 10.1186/s12912-025-03056-2, PMID: 40247325 PMC12004616

[23] Cheng K Xu Z Zhang Y Chen R Gui L (2025). Construction of an evaluation index system for hospital resilience to emerging infectious diseases: a Delphi study from China. BMC Public Health 25:1337. doi: 10.1186/s12889-025-22549-y, PMID: 40211192 PMC11983733

[24] Jiang B Liu H Liu Y Tian Y Nie M Jing W (2024). Development of an early mobilization practice for critically ill patients in intensive care units: a Delphi method study. Am J Transl Res 16:3875–85. doi: 10.62347/ASAB6587, PMID: 39262699 PMC11384373

[25] Lam K Abràmoff MD Balibrea JM Bishop SM Brady RR Callcut RA (2022). A Delphi consensus statement for digital surgery. NPJ Digit Med 5:100. doi: 10.1038/s41746-022-00641-6, PMID: 35854145 PMC9296639

[26] Liu Y Eckert CM Earl C (2020). A review of fuzzy AHP methods for decision-making with subjective judgements. Expert Syst Appl 161:113738. doi: 10.1016/j.eswa.2020.113738

[27] Wang B Sun W Liu F Zheng T Quan L Liu G (2025). Construction of a sensitive index system for nursing quality in the perioperative period of liver transplantation based on the three-dimensional quality structure theory. BMC Health Serv Res 25:510. doi: 10.1186/s12913-025-12676-y, PMID: 40197518 PMC11977936

[28] Pant S Garg P Kumar A Ram M Kumar A Sharma HK (2024). AHP-based multi-criteria decision-making approach for monitoring health management practices in smart healthcare system. Int J Syst Assur Eng Manag 15:1444–55. doi: 10.1007/s13198-023-01904-5

[29] Paige SR Miller MD Krieger JL Stellefson M Cheong J (2018). Electronic health literacy across the lifespan: measurement invariance study. J Med Internet Res 20:e10434. doi: 10.2196/10434, PMID: 29986848 PMC6056742

[30] Kickbusch I Piselli D Agrawal A Balicer R Banner O Adelhardt M (2021). The lancet and financial times commission on governing health futures 2030: growing up in a digital world. Lancet 398:1727–76. doi: 10.1016/S0140-6736(21)01824-9, PMID: 34706260

[31] Paige SR Stellefson M Krieger JL Miller MD Cheong J Anderson-Lewis C (2019). Transactional eHealth literacy: developing and testing a multi-dimensional instrument. J Health Commun 24:737–48. doi: 10.1080/10810730.2019.1666940, PMID: 31583963 PMC7366705

[32] Ivanitskaya L O’Boyle I Casey AM (2006). Health information literacy and competencies of information age students: results from the interactive online research readiness self-assessment (RRSA). J Med Internet Res 8:e6. doi: 10.2196/jmir.8.2.e6, PMID: 16867969 PMC1550696

[33] Yoon J Lee M Ahn JS Oh D Shin S-Y Chang YJ (2022). Development and validation of digital health technology literacy assessment questionnaire. J Med Syst 46:13. doi: 10.1007/s10916-022-01800-8, PMID: 35072816 PMC8784987

[34] Negera D Zewdie A Kera AM Degefa GH (2023). Health information use and associated factors among healthcare professionals in ilu aba bor zone, Oromia region, Ethiopia: an institution-based cross-sectional study. BMJ Open 13:e067540. doi: 10.1136/bmjopen-2022-067540, PMID: 36914187 PMC10016269

[35] van der Vaart R Drossaert C (2017). Development of the digital health literacy instrument: measuring a broad spectrum of health 1.0 and health 2.0 skills. J Med Internet Res 19:e27. doi: 10.2196/jmir.6709, PMID: 28119275 PMC5358017

[36] Ernstmann N Halbach S Kowalski C Pfaff H Ansmann L (2017). Measuring attributes of health literate health care organizations from the patients’ perspective: development and validation of a questionnaire to assess health literacy-sensitive communication (HL-COM). Z Evid Fortbild Qual Gesundhwes 121:58–63. doi: 10.1016/j.zefq.2016.12.008, PMID: 28545615

[37] Norman CD Skinner HA (2006). eHealth literacy: essential skills for consumer health in a networked world. J Med Internet Res 8:e9. doi: 10.2196/jmir.8.2.e9, PMID: 16867972 PMC1550701

[38] Gao Y Yan K Yan X Xi N Gao J Ren H (2023). Correlation between health literacy and health-related quality of life in patients with diabetic peripheral neuropathy: the mediating role of self-management. Nurs Open 10:3164–77. doi: 10.1002/nop2.1566, PMID: 36572957 PMC10077377

[39] Wang C Kane RL Xu D Meng Q (2015). Health literacy as a moderator of health-related quality of life responses to chronic disease among chinese rural women. BMC Womens Health 15:34. doi: 10.1186/s12905-015-0190-5, PMID: 25887361 PMC4399716

[40] Wieczorek M Meier C Vilpert S Reinecke R Borrat-Besson C Maurer J (2023). Association between multiple chronic conditions and insufficient health literacy: cross-sectional evidence from a population-based sample of older adults living in Switzerland. BMC Public Health 23:253. doi: 10.1186/s12889-023-15136-6, PMID: 36747134 PMC9901105

[41] Liu X Gan X Ren G Mao Z Hu J Sha C (2025). Path analysis of the influence of digital health literacy on self-management behaviour among elderly patients with chronic diseases in rural China. BMC Geriatr 25:293. doi: 10.1186/s12877-025-05952-3, PMID: 40301797 PMC12039138

[42] Dijkman EM Ter Brake WWM Drossaert CHC Doggen CJM (2023). Assessment tools for measuring health literacy and digital health literacy in a hospital setting: a scoping review. Healthc (Basel Switz) 12:11. doi: 10.3390/healthcare12010011 PMC10778720 38200917

[43] Kim S Austin L Liu BF Jin Y (2022). Exploring differences in crisis literacy and efficacy on behavioral responses during infectious disease outbreaks. Public Relat Rev 48:102204. doi: 10.1016/j.pubrev.2022.102204

[44] Ziapour A Malekzadeh R Darabi F Yıldırım M Montazeri N Kianipour N (2024). The role of social media literacy in infodemic management: a systematic review. Front Digit Health 6:1277499. doi: 10.3389/fdgth.2024.1277499, PMID: 38419808 PMC10899688

[45] Council recommendation on key competences for lifelong learning - European education area. (2022). Available online at: https://education.ec.europa.eu/focus-topics/improving-quality/key-competences (Accessed June 24, 2025)

[46] Tinmaz H Lee Y-T Fanea-Ivanovici M Baber H (2022). A systematic review on digital literacy. Smart Learn Environ 9:21. doi: 10.1186/s40561-022-00204-y, PMID: 40478098 PMC9175160

[47] Li F Cheng L Wang X Shen L Ma Y Islam AYMA (2025). The causal relationship between digital literacy and students’ academic achievement: a meta-analysis. Humanit Soc Sci Commun 12:108. doi: 10.1057/s41599-025-04399-6

[48] Price-Haywood EG Arnold C Harden-Barrios J Davis T (2023). Stop the divide: facilitators and barriers to uptake of digital health interventions among socially disadvantaged populations. Ochsner J 23:34–42. doi: 10.31486/toj.22.0101, PMID: 36936477 PMC10016217

